# Low mood in a sample of 5–12 year-old child psychiatric patients: a cross-sectional study

**DOI:** 10.1186/s13034-017-0183-2

**Published:** 2017-10-06

**Authors:** Katri Maasalo, Jaana Wessman, Eeva T. Aronen

**Affiliations:** 1 University of Helsinki and Helsinki University Hospital, Children’s Hospital, Child Psychiatry, Tukholmankatu 8 C 613, 00290 Helsinki, Finland; 2 Helsinki Pediatric Research Center, Laboratory of Developmental Psychopathology, Helsinki, Finland

**Keywords:** Low mood, Behavioural problems, Global functioning

## Abstract

**Background:**

Not much is known about low mood and its associates in child psychiatric patients. In this study, we examined the prevalence of low mood, how it associates with disruptive behaviour, and affects clinician-rated global functioning in child psychiatric outpatients.

**Methods:**

The study population consisted of 862 5–12 year-old child psychiatric patients. The study sample was a subsample of all 1251 patients attending a child psychiatric outpatient clinic at Helsinki University Hospital in 2013–2015 formed by excluding 4 year-old and 13 year-old patients and those with missing or incomplete data. The parent-rated Strengths and Difficulties Questionnaire, collected as part of the routine clinical baseline measure, was used as a measure of psychiatric symptoms. The diagnoses were set according to ICD-10 by the clinician in charge after an initial evaluation period. The Children’s Global Assessment Scale (CGAS) score set by clinicians provided the measure of the patients’ global functioning. All information for the study was collected from hospital registers. Associations between emotional symptoms and conduct problems/hyperactivity scores were examined using ordinal regression in univariate and multivariate models, controlling for age and sex. The independent samples T test was used to compare the CGAS values of patient groups with low/normal mood.

**Results:**

In our sample, 512 children (59.4%) showed low mood. In multivariate ordinal regression analysis, low mood associated with conduct problems (OR 1.93, 95% CI 1.39–2.67), but no association was found between low mood and hyperactivity. Low mood was prevalent among children with oppositional defiant disorder or conduct disorder (51.8%). The global functioning score CGAS was lower among children with parent-reported low mood (52.21) than among children with normal mood (54.62, p < 0.001). The same was true in the subgroup of patients with no depression diagnosis (54.85 vs. 52.82, p = 0.001).

**Conclusions:**

Low mood is prevalent in child psychiatric outpatients regardless of depression diagnosis and it has a negative effect on global functioning. Low mood and behavioural problems are often associated. It is important to pay attention to low mood in all child psychiatric patients. We recommend prevention measures and low-threshold services for children with low mood.

## Background

Among children, the coexistence of psychiatric symptoms across diagnostic categories is a rule rather than an exception [1, 2]. Emotional and behavioural symptoms tend to overlap in population-based samples [2–4], and comorbidity is also common among child psychiatric patients [5, 6]. In order to better “convey the mixed patterns of symptomatology” [1] that are common in child psychiatry, a combination of dimensional and categorical approaches to diagnosis is recommended.

As the evidence of the clinical significance of subthreshold symptoms has grown, many studies support a dimensional rather than categorical view of disorders, also in depressive disorders [7–9]. Subthreshold symptoms of depression impair quality of life and global functioning, and pose a risk of future psychopathology. There is evidence that recognizing and treating them has clinical significance [7, 8], but further studies in clinical child populations are needed.

In our previous study in a Finnish non-clinical population of 4–12 year-old children, emotional problems were associated with conduct problems and hyperactivity, and our findings emphasized the role of low mood in the associations between emotional and behavioural problems [10]. Persistent sad or low mood is one of the core symptoms of depression according to both the DSM-5 [11] and ICD-10 [12] classification systems, and is also a common symptom of subthreshold depression [13, 14]. Some studies on the clinical characteristics of youth with depression report rates of low mood ranging from 50.0 to 100% [14–18]. One study [15] has compared the prevalence of depressive symptoms in depressed (98.2–100%) and other adolescent psychiatric patients (2.9–4.1%). A recent Danish population-based study found low mood to be as frequent among 8–10 year-old children with subthreshold depression as among those with clinical depression (94.5% vs. 94.3%; diagnoses from DAWBA entered online by mothers and reviewed by physicians), and distinctly less common although still quite prevalent (16.4%) among non-depressed children [13]. In our population-based study of Finnish children [10], low mood was reported by 16% of the children’s parents. Low mood was associated with family structure, sleep problems, illness or disability of the child, conduct problems, and hyperactivity. Examining low mood at symptom level and how it associates with behavioural symptoms and disorders in a sample of child psychiatric patients is important, as this knowledge deepens the understanding of the relations between mood and behavioural problems in child patients. This knowledge also has relevance for diagnostic decisions and choices of treatment options.

Children with irritable mood (which counts as a symptom of both depression and mania in children) have been the object of vast interest and several studies, partly in a response to diagnosing children with bipolar disorder [19]. Recently, a new diagnosis of disruptive mood dysregulation disorder (DMDD) has been introduced to the DSM-5 [11]. This new mood diagnosis, and the fact that bipolar disorder remains an inadequately understood disorder in children, calls for studies of co-occurrence of mood and behavioural problems in clinical samples.

We found no earlier literature on studies on comorbidity in clinical populations where the presence of emotional and behavioural symptoms is considered without being restricted into diagnostic categories. Studies on low mood are also scarce—we found no studies that report rates of low mood in child psychiatric patients other than those with depression. Further, no studies were found to examine the associations between low mood and externalising behaviour in child psychiatric patients.

Our aim in this study was to evaluate how emotional symptoms, especially low mood and behavioural problems, coexist in a sample of 5–12 year-old child psychiatric outpatients. More specifically, we wanted to examine how conduct problems/hyperactivity associate with emotional symptoms, the prevalence of low mood in different patient groups, how parents’ and the children’s reports on mood correspond with each other, how low mood associates with disruptive behaviour, and how low mood affects the clinician-rated global functioning of the child. On the basis of our previous results [10], we hypothesized that emotional problems and low mood would be associated with conduct problems and hyperactivity. We also hypothesized that low mood would more frequently be reported by children than their parents, and that it would have a negative effect on the clinician-rated global functioning of the child.

## Methods

Our study population consisted of 862 5–12 year-old child psychiatric patients. We formed the study sample from all the 1251 patients who attended the child psychiatric assessment and acute care unit of Helsinki University Hospital in 2013–2015 by excluding the few 4 year-old and 13 year-old patients and those with missing or incomplete data on parent reported psychiatric symptoms. The final study population did not differ from the initial patient population in respect to age, sex or CGAS values.

The information for the study was collected from hospital registers. Strengths and Difficulties Questionnaire (SDQ-parent form) and Quality of Life measure (17D-child report) were collected as a routine clinical baseline measure of the child’s psychiatric symptoms and quality of life at Helsinki University Hospital Child Psychiatry Clinic.

A clinician set the diagnoses according to ICD-10 and assigned the CGAS values after an initial evaluation. The initial evaluation included the information from the referral, a meeting with the child and the parents where the anamnesis was taken by the child psychiatrist, and a brief discussion with the parents alone and with the child alone. The researchers divided the detailed diagnoses (e.g. mild, moderate, severe major depressive disorder) into diagnostic groups (e.g. depressive disorder) by assigning a group to each ICD-10 diagnose code in the data. The CGAS [20] was used as a measure of patients’ global functioning, the scale of which ranges from 0 to 100; higher scores indicating better functioning. Our clinic routinely uses this scale which has shown to have moderate inter-rater validity in a naturalistic clinical setting [21].

The SDQ is a brief 25-item instrument for screening the emotional and behavioural problems of children and adolescents [22]. The items are scored 0/1/2 for “not/somewhat/certainly true”, except for 5 items (items 7, 11, 14, 21 and 25) that are scored in the opposite direction. The items are categorized into emotional problems, conduct problems, hyperactivity, peer problems, and prosocial subscales, with scores ranging from 0 to 10. A total score of 0–40 is generated by summing the scores of the four first-mentioned subscales [23]. Epidemiological studies [24] have shown the SDQ to be applicable to Finnish children. The SDQ subscores were categorized as “normal”, “borderline” or “abnormal” using the cut-off points defined on the official SDQ website [23] (0–3, 4, 5–10 for emotional problems, 0–2, 3, 4–10 for conduct problems, and 0–5, 6, 7–10 for hyperactivity). Of the items screening for emotional problems, “often unhappy, down-hearted or tearful” directly describes mood, while the others describe anxiety symptoms and somatic complaints. We used mood item number 13 as a measure for mood as rated by parents in the sample, and a depression dimension (question 17) from the 17D as a measure for mood as reported by children. The 17D is a 17-dimensional, generic measure of perceived health-related quality of life for pre-adolescents [25]. Question 17 asks the child to choose whether they feel cheerful and happy or a little/quite/very/extremely sad, unhappy or depressed. Reports of feeling at least a little sad, unhappy or depressed were interpreted as current low mood.

### Statistical analyses

The “somewhat true” and “certainly true” categories of the emotional items of the SDQ were collapsed into a “somewhat or certainly true” category to retain the setting that was used in our population-based study [10] and to add sensitivity to the parents’ reports, since parents often underestimate children’s internalizing symptoms [26–29]. The associations between emotional symptoms and conduct problem/hyperactivity scores were examined using ordinal regression in univariate and multivariate models. The kappa statistic (presented in the results) was used to assess the level of agreement between parents and children on mood. The independent samples T test was used to compare the CGAS values of patient groups.

Analyses were carried out using IBM SPSS Statistics 22.

## Results

### Descriptive statistics

Table 1 presents the characteristics and clinical diagnoses of the study population as well as the distribution of the scores on SDQ subscales and emotional problems subscale items. In the SDQ, boys had higher total difficulties scores as well as higher conduct problems and hyperactivity subscale scores than girls, whereas girls had higher emotional problems scores (p = 0.000–0.004). There was no difference between sexes in peer problems scores. Emotional problems increased with age (p = 0.002), whereas the total problems score, hyperactivity score and conduct score decreased with increasing age (p < 0.001). Age had no effect on peer problems or prosocial scores.

**Table 1:** Descriptive statistics (n = 862)

		Diagnosis, n (%)	
Age in years, mean (SD)	9.1 (2.0)	ODD/CD	224 (26.0)
Range	5–12	Hyperkinetic disorder	152 (17.6)
Interquartile range	4	Other emotional diagnoses	136 (15.8)
Preschool age, n (%)	105 (12.2)	Anxiety disorder	114 (13.2)
School age, n (%)	757 (87.8)	Depression	99 (11.5)
Girls, n (%)	313 (36.3)	Learning disability	70 (8.1)
Boys, n (%)	549 (63.7)	Post-traumatic disorder	61 (7.1)
		Autism spectrum disorder	57 (6.6)
CGAS on arrival (n = 849)		Somatic diagnosis	31 (3.6)
Mean (SD)	53.2 (8.1)	Obsessive compulsive disorder	23 (2.7)
Median	52.0	Eating disorder	23 (2.7)
Range	21–92	Sleeping problem diagnosis	21 (2.4)
Interquartile range	12	Other diagnosis	115 (13.3)

The proportions of SDQ scores	Normal, n (%)	Borderline, n (%)	Abnormal, n (%)
Total difficulties score	306 (35.5)	138 (16.0)	418 (48.5)
Emotional problems score	380 (44.1)	113 (13.1)	369 (42.8)
Conduct problems score	300 (34.8)	137 (15.9)	425 (49.3)
Hyperactivity score	471 (54.6)	75 (8.7)	316 (36.7)
Peer problems score	362 (42.0)	145 (16.8)	355 (41.2)
Prosocial score	547 (63.5)	130 (15.1)	185 (21.5)

The proportions of scores on emotional problems subscale	Not true, n (%)	Somewhat true, n (%)	Certainly true, n (%)
Often complains of headaches, stomach-aches or sickness	364 (42.2)	317 (36.8)	174 (20.2)
Many worries, often seems worried	282 (32.7)	376 (43.6)	202 (23.4)
Often unhappy, down-hearted or tearful	350 (40.6)	364 (42.2)	148 (17.2)
Nervous or clingy in new situations, easily loses confidence	310 (36.0)	327 (37.9)	221 (25.6)
Many fears, easily scared	377 (43.7)	330 (38.3)	153 (17.7)

*SD* standard deviation, *CGAS* the Children’s Global Assessment Scale, *ODD* oppositional defiant disorder, *CD* conduct disorder, *SDQ* the Strengths and Difficulties Questionnaire

### Emotional problems, conduct problems and hyperactivity scores in the SDQ

**Fig. 1:**
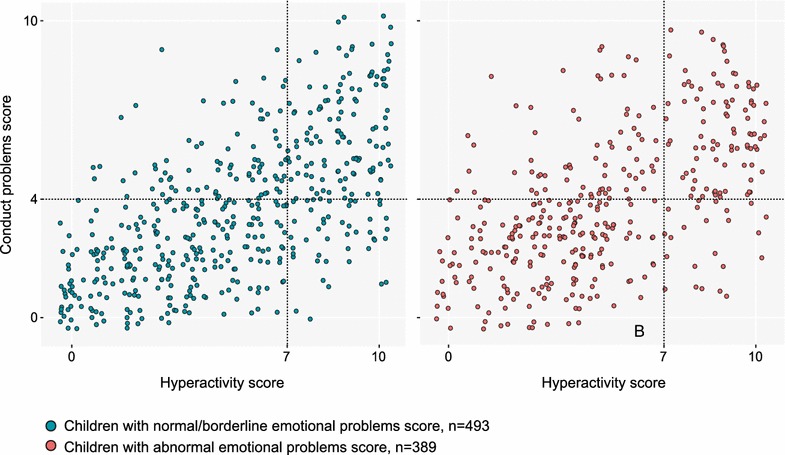
Emotional problems, conduct problems and hyperactivity according to parent-rated SDQ. The figure shows the distribution of emotional problems scores, conduct problems scores and the hyperactivity scores in the parent-rated SDQ. The emotional problems are presented as categories of children with normal/borderline emotional problems score on the *left* and the children with abnormal emotional problems score on the *right*. The conduct problems score and the hyperactivity score are presented on a continuous scale, with the abnormal cut-off point marked by the *dashed line*. In our sample, 24.4% of the children scored below the cut-off point in all three scales. Of the children with abnormal conduct problems and/or hyperactivity scores, 42.2% also had an abnormal emotional problems score. 18.8% of the patients only had an abnormal emotional problems score, with no conduct problems/hyperactivity. The smallest patient group (11.7%) was that of children with emotional problems and hyperactivity but without conduct problems was the smallest patient group

The partial correlation (controlling for age and sex) between the emotional problems score and the conduct problems score in the parent-rated SDQ was 0.124 (p < 0.001), between the emotional problems score and the hyperactivity score 0.052 (p = 0.129), and between the conduct problems score and the hyperactivity score 0.574 (p < 0.001). Of the children with abnormal conduct problems and/or hyperactivity scores, 42.2% also had an abnormal emotional problems score, and 162 (18.8%) of the patients had only an abnormal emotional problems score, with no conduct problems/hyperactivity. Of the patients, 101 (11.7%) had abnormal scores in all three categories, and 210 patients (24.4%) scored under the cut-off point in all three scales. The smallest patient group was that of children with hyperactivity and emotional problems but no conduct problems (n = 21, 2.4%). See also Fig. 1.

### Low mood reported by parents and children

In our sample, 512 children (59.4%) showed low mood (defined as SDQ item 13 “Often unhappy, down-hearted or tearful” being rated somewhat or certainly true by a parent). Of the 428 children who responded to the 17D mood question, 48.8% reported feeling at least a little sad, unhappy or depressed and 62.1% were evaluated to have low mood by their parent. In 166 cases, both the child and the parent reported low mood, and in 119 cases, both the child and the parent reported normal mood. In 23.4% of the cases the parent reported low mood although the child did not, and in 10.0% of the cases the situation was vice versa. The parent and child agreed on the child’s mood in 66.6% of the cases, and disagreed in 33.4% of the cases. Cohen’s kappa for agreement on mood between parent and child was 0.336 (p < 0.001, 95% CI 0.250–0.422). The majority of the children with parent-reported low mood (58.4%) were boys, as were those with self-reported low mood (55.0%). As the girls were the minority in the whole sample (36.3%), they were over-represented in these groups, making both self-reported and parent-reported low mood more common among girls (59.1% in girls vs. 42.8% in boys, and 68.1% in girls vs. 54.5% in boys, respectively).

### Relationship between mood, conduct problems and hyperactivity

In univariate ordinal regression analysis of emotional symptoms and behavioural problems (controlling for age and sex), low mood, worrying, and somatic complaints were associated with conduct problems. The strongest association was between mood and conduct problems (OR 2.03, 95% CI 1.55–2.66). In multivariate analysis, low mood remained the only associate with conduct problems (OR 1.93, 95% CI 1.39–2.67). No association was found between emotional symptoms and hyperactivity. The results are presented in Table 2.

**Table 2:** The association of emotional symptoms with conduct problems and hyperactivity in child psychiatric patients (n = 862)

	Conduct problems score		Hyperactive score	
	Univariate	Multivariate	Univariate	Multivariate
	OR (95% CI)	OR (95% CI)	OR (95% CI)	OR (95% CI)
Often unhappy, down-hearted or tearful	2.03 (1.55–2.66)***	1.93 (1.39–2.66)***	0.98 (0.74–1.30)	0.97 (0.70–1.36)
Often complains of headaches, stomach aches or sickness	1.38 (1.05–1.80)*	1.10 (0.83–1.48)	1.10 (0.83–1.45)	1.13 (0.83–1.53)
Many worries, often seems worried	1.50 (1.14–1.98)**	1.12 (0.79–1.59)	0.98 (0.74–1.30)	0.99 (0.69–1.42)
Nervous or clingy in new situations, easily loses confidence	1.21 (0.92–1.58)	1.04 (0.78–1.39)	1.11 (0.84–1.49)	1.16 (0.86–1.58)
Many fears, easily scared	1.19 (0.91–1.54)	0.85 (0.62–1.16)	0.88 (0.67–1.16)	0.82 (0.60–1.14)

Regression analysis examining the problem scores as explained variables and emotional symptoms as predictor variables. The problem scores were categorized as normal, borderline or abnormal (0–2; 3; 4–10 for conduct problems and 0–5; 6; 7–10 for hyperactivity) and the emotional symptoms dichotomized in “not true” and “somewhat/certainly true” (controlling for age and sex)

*OR* odds ratio, *CI* confidence interval

* p < 0.05, ** p < 0.01, *** p < 0.001

Table 3 presents the proportions of children with normal/low mood relative to other conditions. Of the children who scored within the abnormal range of the conduct problems score, 64.5% also showed parent-reported low mood, and the same was true for 56.0% of the children who scored within the abnormal range of the hyperactivity score. Of the 251 children (29.1% of the whole sample) who scored within the abnormal range in both conduct problems and hyperactivity scales, 60.6% also showed parent-reported low mood (n = 152, 17.6% of the whole sample). Of the 99 children with a depression diagnosis, 81.8% had low mood (48.5% scoring somewhat true, and 33.3% scoring certainly true) according to their parents. Of the children without depression, 56.5% had low mood. The frequency of low mood was 51.8% among the 224 children diagnosed with ODD/CD, and 39.5% among the 152 children diagnosed with a hyperkinetic disorder, the item “often unhappy, down-hearted or tearful” being rated “certainly true” by 15.6 and 7.2% of the children, respectively, and when children with comorbid depression diagnosis were excluded, 14.9 and 6.2% respectively.

**Table 3:** The prevalence of low mood in different patient groups (whole sample, n = 862)

	Normal mood	Low mood	*χ* ^2^	*df*	*p*
Whole sample	350 (40.6)	512 (59.4)			
Children with abnormal conduct problems score	151 (35.5)	274 (64.5)	16.849	2	<0.001
Children with borderline conduct problems score	49 (35.8)	88 (64.2)			
Children with normal conduct problems score	150 (50.0)	150 (50.0)			
Children with abnormal hyperactivity score	139 (44.0)	177 (56.0)	2.952	2	0.23
Children with borderline hyperactivity score	32 (42.7)	43 (57.3)			
Children with normal hyperactivity score	179 (38.0)	292 (62.0)			
Children with no depression	332 (43.5)	431 (56.5)			
Children with depression	18 (18.2)	81 (81.8)			
Children with CD/ODD	108 (48.2)	116 (51.8)			
Children with hyperactive disorder	92 (60.5)	60 (39.5)			

*ODD* oppositional defiant disorder, *CD* conduct disorder, *χ* ^*2*^ Chi square, *df* degrees of freedom, *p* p value

### Mood and global functioning

The effects of mood on global functioning are presented in Table 4. Global functioning rated by CGAS was lower among children with parent-reported low mood (52.21) than among those with normal mood (54.62, p < 0.001). This effect on global functioning remained when clinically depressed children were excluded from the analysis (52.82 vs. 54.85, p < 0.01). CGAS was also lower in children with self-reported low mood than in those with normal mood (52.36 vs. 55.82 respectively, p < 0.001). Children with a depression diagnosis from a clinician had lower global functioning (49.26) than children with no depression diagnosis (53.64, p < 0.001).

**Table 4:** The comparison of global functioning between patient groups

	CGAS
	Mean (SD)
Children with parent-reported low mood (whole sample)	52.21 (7.73)
Children with parent-reported normal mood (whole sample)	54.62 (8.47)***
Children with parent-reported low mood (non-depressed)	52.82 (7.72)
Children with parent-reported normal mood (non-depressed)	54.85 (8.42)**
Children with self-reported low mood	52.36 (7.21)
Children with self-reported normal mood	55.82 (8.49)***
Children with abnormal emotional problems score	52.20 (7.73)
Children with normal or borderline emotional problems score	53.93 (8.83)**
Children with depression	49.25 (7.25)
Children with no depression	53.71 (8.10)***
Children with abnormal hyperactivity score + low mood	51.66 (7.19)
Children with abnormal hyperactivity score + normal mood	53.06 (7.69)
Children with abnormal conduct problems score + low mood	51.60 (7.30)
Children with abnormal conduct problems score + normal mood	52.31 (6.94)
Children with abnormal emotional problems score + low mood	52.08 (7.77)
Children with abnormal emotional problems score + normal mood	53.52 (7.28)

*CGAS* Children’s Global Assessment Scale, *SD* standard deviation

** p < 0.01, *** p < 0.001, from T test

## Discussion

In this study, we examined the prevalence of low mood, how the parents’ and the children’s reports on mood correspond with each other, how low mood associates with behavioural problems, and how low mood affects the clinician-rated global functioning in a sample of 5–12 year-old child psychiatric outpatients.

In our sample, parents reported low mood in 59.4% of the patients. We found no studies that report the prevalence of low mood in child psychiatric patients as such. Instead, studies seem to have examined the clinical characteristics of children and adolescents with depression, and have reported rates of low mood among youths with depression as ranging from 50.0 to 100% [14–18]. Bennet et al. [14] also compared the frequency of depressive symptoms of depressed and other adolescent psychiatric patients. In their clinical control group, low mood was present in 4.1% of the boys and 2.9% of the girls, which is far less than the 56.5% in our sample. This could be due to the different age range of the study participants or to methodological differences. Our sample was younger, and it is possible that with increasing age, symptoms become more specific and better fit the diagnostic categories. In addition, Bennet et al. used the 17-item Depression Rating Scale extracted from the clinician-administered K-SADS interview as a measure of mood, which required at least mild severity, and we used parent/child questionnaires to estimate low mood. Interestingly, Bennet et al. also presented the highest rates available for the frequency of low mood among depressed patients (98.2–100%) and among those with minor depression and dysthymic disorder (100%), which are higher than our rate of low mood in depression (81.8%), suggesting that our definition for low mood was not overly sensitive. Moreover, the rates presented by Bennet et al. are low compared to the study of Wesselhoeft et al. [13], in which 16% of the non-clinical population with no depression or subthreshold depression presented low mood.

To compare the children’s and parents’ reports of depression, we used a different measure for the children (i.e. not the SDQ), as the 17D response was available for a bigger group of patients (428 vs. 132). To assess the degree of agreement we used Cohen’s Kappa, which was 0.336 in this sample. According to widely-used guidelines, values in the range of 0.21–0.4 are considered “fair” agreement, while only values above 0.6 would be considered substantial [30]. Multiple studies have shown that parents’ and children’s agreement on the child’s symptoms is moderate at best [26, 28, 31–33]. In the study of Angold and colleagues [26], 7–25 year-old children and adolescents reported more depressive symptoms than their parents, and agreement was moderate (K = 0.40). Even very low levels of agreement between child and parent regarding the child’s feelings of depression have been reported in 6–12 year-old children (0.03 in a community sample and 0.06 in clinical sample) [34]. In our sample, parents recognized the child-reported low mood in about 80% of the children who reported low mood themselves, but 37.6% of the children with parent-reported low mood reported normal mood. In our study, parents reported more low mood than the children, contrary to some earlier statements that children report more internalizing symptoms than their parents [32, 35, 36]. In our study, evaluation of the presence of low mood was made using only one question, and with different measures for children and parents. While the SDQ covers the last 6 months and is a better measure for sustained low mood, the 17D only asks about current feelings, capturing transitory feelings of low mood but missing low mood in children who momentarily are not feeling sad. Parents may also over-report low mood in children because of their own worries or problems [37]. In clinical samples of chronic somatic disorders, adolescents themselves have reported significantly less depression symptoms than their parents [38, 39]. On the other hand it is possible that it is difficult for child psychiatric patients to always recognize their mood symptoms, or to reveal them. Our results and those mentioned above emphasize the importance of asking both the child and the parent about internalizing symptoms, especially in clinical samples.

In a retrospective chart review study on 75 6–17 year-old youths with depression by Breton et al. the reason for consultation in 28% of the youths (and even 59% of the boys 6–12 years old) were behavioural problems [15]. In our previous study of a non-clinical population [10], low mood was the emotional symptom that associated most with conduct problems. We also found this association between low mood and the abnormal conduct problems score in the parent-rated SDQ in the present clinical sample, though it was not as strong as that in the population sample. Of the children with clinician-diagnosed ODD/CD, 7.1% were diagnosed with comorbid depression. Interestingly, more than half of the children with ODD/CD were reported as having low mood, and 14.9% of the children with ODD/CD but without depression rated the “often unhappy, downhearted or tearful” item as certainly true. Non-clinical samples have shown comorbidity rates of 0–45.9% for depression in children with ODD/CD [2] and clinical samples have shown rates of 10.0–50.0% [17, 40, 41]. A similar prevalence has been reported for comorbidity between subthreshold depression and ODD/CD [8, 13]. We found no earlier studies reporting the prevalence of low mood in children with ODD/CD for comparison. Do these children with low mood and conduct problems have a comorbid state of subthreshold depressive disorder and ODD/CD that meets the criteria for a categorical diagnosis (heterotypic comorbidity)? Or are they children with a depressive disorder presented with irritability, thus misconstrued as a conduct disorder (artificial comorbidity)? Do they represent a totally distinct patient group with a disruptive mood dysregulation disorder? More studies are clearly needed on the associations between low and irritable mood and conduct problems in clinical samples to address these questions.

Contrary to our finding in the non-clinical population, we found no association between mood and hyperactivity in parent-rated SDQ. However, over one-third of the children with a diagnosed hyperkinetic disorder had low mood. The reported comorbidity rates of depression in children with ADHD range from 0 to 75% [2, 42, 43]. A recent meta-analysis [44] on the correlations between ADHD and depression reported mixed evidence on the associations of the two disorders. The overall meta-analysis resulted in a moderate association, but there was heterogeneity across studies, and certain subgroup analyses resulted in small or unreliable associations. In a study by Elia et al. [45], minor depression/dysthymia (MDDD) was among the most common comorbidities in youths with ADHD (21.6%). It also found that 10.8% of children with ADHD met the criteria for simultaneous ODD, MDDD and combined type ADHD. Most of these children had irritability as a symptom, and accounted for nearly half of the children with irritability in the whole study population. Irritability is a mood state; it is closely related to low mood but is also an externalizing symptom that makes the child prone to anger and temper outbursts [19, 46]. It seems to predict future depression and anxiety, but not CD or ADHD at follow-up [19].

As recently reviewed by Zisner and Beauchaine [47], shared mechanisms of neural dysfunction in dopaminergic mesolimbic circuits associated with irritability, anhedonia and impulsive behaviour could in part account for the comorbidity patterns between depression and externalizing symptoms.

The finding that almost a quarter (24.4%) of the patients had no abnormal emotional problems, conduct problems or hyperactivity scores in the SDQ is somewhat surprising for patients in a tertiary clinic, but a Chinese study also reported similar findings, in which only half (51% when parent rated and 52% when self rated) of the adolescents scored within the abnormal range of the SDQ total problems score [48]. Our study population most likely also includes a group of children who only have abnormal peer problems scores not examined in this study, so that the number of children with no abnormal scores in any problems subscales of the SDQ is probably at least a little smaller than the 24.4% above. The problems in ADD without hyperactivity, and autism spectrum disorders with mild severity may not fall into SDQ problems categories or may be limited to peer problems. Moreover, as taken into account in the algorithms when predicting psychiatric diagnosis from SDQ, even scores below the abnormal cut-off points are of clinical relevance when combined with symptoms that impact the child’s everyday life.

Low mood according to either parent or child lowered the global functioning of the child, implying that recognition of low mood is important. This was true even in children without a depression diagnosis, which is in line with the findings that subthreshold depression affects the quality of life and performance [8]. In addition, children with low mood and either conduct problems or hyperactivity had lower CGAS values than the children with normal mood, but this difference did not reach statistical significance. This can be interpreted to mean that behavioural problems in children with an abnormal hyperactivity or conduct problems score are more relevant in respect to global functioning.

It is important to view these results in the light of certain limitations of this study. As the data were cross-sectional, no conclusions can be made on the longitudinal associations of the co-occurring symptoms or of low mood and global functioning. In addition, we can only state that children with low mood have poorer global functioning than children with normal mood; we cannot claim that low mood is the reason for the decline. It can be speculated that the opposite could also be true: that children feel sad or unhappy if they are unable to function normally. We used diagnoses set by clinicians according to ICD-10, based on clinical information collected during the initial assessment of the children. The diagnoses for the patients were compiled from medical records. As no structured diagnostic interviews were conducted, some of the co-occurring problems may have remained unnoticed by clinicians, and thus not diagnosed.

According to our results, low mood is a common symptom in children first coming to a child psychiatric clinic—in children with depression as well as with behavioural problems. In clinical practice the importance of careful assessment to define the temporal relationship of different symptoms to determine the principal target of treatment is pointed out. It has also been suggested that by paying attention to depressive symptoms with children with ODD/CD future depression could be prevented [49] as well as depression and other comorbidities in children with ADHD [50].

According to our results, patients with low mood have lower global functioning than patients with normal mood indicating that these children need special attention. The children with significant depressive symptoms have been seen as a potential object of intervention and secondary prevention decreasing the risk for recurrent depression [51].

## Conclusion

We conclude that it is important to assess mood in all child psychiatric patients and to pay attention to low mood even in the absence of clinical depression. We recommend prevention measures and low-threshold services for children with low mood.

## Abbreviations

ADD: attention-deficit disorder
ADHD: attention-deficit/hyperactivity disorder
CD: conduct disorder
CGAS: The Children’s Global Assessment Scale
CI: confidence interval
DAWBA: the Development and Well-Being Assessment
DMDD: disruptive mood dysregulation disorder
DSM-5: the Diagnostic and Statistical Manual of Mental Disorders, 5th edition
ICD-10: International Statistical Classification of Diseases and Related Health Problems, 10th edition
K-SADS: the Kiddie Schedule for Affective Disorders and Schizophrenia
MDDD: minor depression/dysthymia
ODD: oppositional defiant disorder
OR: odds ratio
SDQ: Strengths and Difficulties Questionnaire
